# Effects of *Cynara scolymus* L. Bract Extract on Lipid Metabolism Disorders Through Modulation of HMG-CoA Reductase, Apo A-1, PCSK-9, p-AMPK, SREBP-2, and CYP2E1 Expression

**DOI:** 10.3390/metabo14120728

**Published:** 2024-12-23

**Authors:** Imane Mokhtari, Abdelaaty A. Shahat, Omar M. Noman, Dragan Milenkovic, Souliman Amrani, Hicham Harnafi

**Affiliations:** 1 Laboratory of Bioresources, Biotechnologies, Ethnopharmacology and Health, Faculty of Sciences, University Mohamed I, Oujda 60000, Morocco; mokhtari.imane@ump.ac.ma (I.M.); samrani@ump.ac.ma (S.A.); 2 Department of Pharmacognosy, College of Pharmacy, King Saud University, P.O. Box 2457, Riyadh 11451, Saudi Arabia; ashahat@ksu.edu.sa (A.A.S.); onoman@ksu.edu.sa (O.M.N.); 3 Plants for Human Health Institute, Department of Food, Bioprocessing and Nutrition Sciences, North Carolina State University, Kannapolis, NC 28081, USA; dmilenkovic@ucdavis.edu

**Keywords:** *Cynara scolymus*, lipid metabolism, HMG-CoA reductase, SREBP-2, p-AMPK

## Abstract

**Background/Objectives:** Hyperlipidemia is a major contributor to metabolic complications and tissue damage, leading to conditions such as liver steatosis, atherosclerosis, and obesity. This study aimed to investigate the effects of aqueous artichoke bract extract (AE) on lipid metabolism, liver antioxidative defense, and liver steatosis in mice fed a high-fat, high-sucrose diet while elucidating the underlying mechanisms. **Methods:** An 8-week study used hyperlipidemic mice treated with AE at daily doses of 100 and 200 mg/kg bw, compared to fenofibrate. Plasma, liver, fecal, and biliary lipids, as well as blood glucose, were analyzed enzymatically. The liver antioxidative defense was assessed by measuring reduced glutathione, malondialdehyde (MDA), and antioxidant enzyme activities, while liver steatosis was evaluated through transaminase and alkaline phosphatase activities and histological monitoring of lipid droplets. Polyphenol profiling and quantification were performed using HPLC–DAD, and potential mechanisms were predicted by molecular docking and confirmed in HepG2 cells. **Results:** At 200 mg/kg, AE significantly improved plasma lipid profiles by reducing total cholesterol, triglycerides, and LDL–cholesterol while increasing HDL–cholesterol. It facilitated cholesterol reduction in the liver and its excretion, indicating activation of reverse cholesterol transport, which led to reduced body weight and liver steatosis. AE lowered MDA levels and enhanced antioxidant enzyme activities. AE was found to be safe (LD50 > 5000 mg/kg) and modulated gene expression in HepG2 cells. **Conclusions:** Based on our results, the artichoke bract extract could be considered a natural resource of bioactive compounds to treat hyperlipidemia and related cardiometabolic diseases.

## 1. Introduction

Cardiovascular diseases remain a leading cause of mortality in Morocco and many other countries worldwide [1]. One of the most critical risk factors for developing these diseases is oxidative stress, which is linked to elevated levels of cholesterol, triglycerides, and LDL–cholesterol, along with decreased levels of HDL–cholesterol [2]. Consequently, managing these lipid parameters is vital for maintaining cardiovascular health. In fact, epidemiological studies have shown a negative correlation between circulating HDL–cholesterol and its main protein component, apoA-1 levels, and cardiovascular diseases [3].

The major protective function of HDL–cholesterol is its role in the reverse cholesterol transport pathway (RTC), where apoA-1 takes up excess cholesterol from peripheral tissue and returns it to the liver for further excretion in bile [4]. The Apo A-1 is predominantly expressed in the liver and was demonstrated to be regulated by the nuclear receptors LXRα and PPARα [5]. Furthermore, the cholesterol homeostasis was also maintained by modulation of HMG-CoA reductase responsible for hepatic cholesterol biosynthesis [6]. The expression of this enzyme was demonstrated to be downregulated by SREBP-1 [7]. On the other hand, it has recently been demonstrated that the catabolism of LDL–cholesterol is closely linked to the expression of the PCSK-9 protein, which reduces the expression of LDL receptors [8]. As a result, PCSK-9 is currently considered a potential target for the development of cholesterol-lowering and antiatherogenic therapies [8]. Moreover, lipid metabolism is significantly regulated by the adenosine monophosphate-activated protein kinase (AMPK) so that the phosphorylation of this enzyme downregulates acetyl-CoA carboxylase and then lipogenesis [9]. Additionally, phosphorylation of AMPK decreases cholesterol levels through inhibition of HMG-CoA reductase activity [9]. Furthermore, it is shown that AMPK restores triglyceridemia and prevents hepatic lipid accumulation in hypercaloric diet-fed mice [10]. Therefore, this protein can constitute a possible target of hypolipidemic and anti-obesity drugs.

Additionally, in addition to hyperlipidemia, oxidative stress and the production of free radicals are recognized as triggers for nonalcoholic fatty liver disease (NAFLD) [11]. In fact, several studies show that mitochondrial dysfunction leads to overexpression of CYP2E1 (Cytochrome P450 2E1), which generates free radicals and reactive oxygen species that promote oxidative stress and lipid peroxidation [12]. This enzyme was demonstrated to be responsible for excessive fat accumulation and worsening oxidative stress by causing inflammation, liver cell damage, and death [12]. Clearly, the search for drugs that inhibit CYP2E1 overexpression is essential for the treatment of NAFLD [13].

Current hypolipidemic medicines have a variety of side effects, like severe muscle damage, liver and kidney dysfunction, and skin dryness [14]. Therefore, there has been a growing interest in exploring the hypolipidemic and antioxidant effects of natural products as alternative approaches [15].

The artichoke (*Cynara scolymus* L.), belonging to the Asteraceae family, originates from the Mediterranean region, including North Africa and Southern Europe, and serves both culinary and medicinal purposes [16]. In Morocco, artichoke extract has been used for its potential benefits in treating digestive disorders and liver diseases and as a remedy for lowering cholesterol [17,18]. Scientific evidence supports these traditional claims, as artichoke extract has demonstrated hepatoprotective, choleretic, and hypolipidemic effects in both clinical and experimental studies [19,20,21,22]. However, the mechanisms of action that may underlie the pharmacological effects described are largely unexplored. These biological activities are most likely associated with the phytochemical profile, including high levels of phenolic compounds [23]. Chlorogenic acid, 1,5-di-*O*-caffeoylquinic acid, and 1,3-di-*O*-caffeoylquinic acid are the major phenolic acids identified in the plant. Other polyphenols, including luteolin, apigenin, and apigenin-7-rutinoside, have been identified in artichoke [24].

This study aims to investigate the effects of an aqueous extract of artichoke bracts on lipid metabolism and liver antioxidative defense in hypercaloric diet-fed mice, focusing on the underlying mechanisms. The research will explore the potential of artichoke extract as a natural alternative to conventional hypolipidemic treatments, with particular emphasis on its impact on lipid levels, oxidative stress, and liver function. In the following sections, we will outline the methodology, present the results, and discuss their significance, highlighting the potential implications for treating hyperlipidemia and related disorders.

## 2. Materials and Methods

### 2.1. Preparation of the Artichoke Bract Extract (AE)

Fresh artichokes (*Cynara scolymus*. L.) were purchased from a biological farm in Berkane City (Morocco) and identified by a taxonomist (Prof. A. Berrichi, Department of Biology, Faculty of Sciences, Oujda, Morocco). A voucher specimen was deposited under the collection number C.S.14. The bracts were manually separated and dried in a ventilated oven at 40 °C for 48 h and then reduced into fine powder. The bract extract was prepared using the same method as that used in traditional medicine, with a few modifications. In brief, the dried powder of artichoke bract (100 g) was infused in the boiling water (500 mL) under sonication for 30 min; the obtained preparation was filtered and dried at 40 °C to obtain a dry powder of aqueous artichoke bract extract (AE), which was conserved at 4 °C until use. The extraction yield was calculated as follows: Yield of extraction (%) = (mass of extract/mass of bract powder) × 100.

### 2.2. Total Polyphenol Content of the AE

The total polyphenol content was determined according to the Folin–Ciocalteu method as previously described [25]. Thus, 0.5 mL of AE was mixed with Folin–Ciocalteu reagent (0.25 mL) and sodium carbonate (0.5 mL). The absorbances were recorded at 725 nm. The concentration was calculated according to a calibration curve of chlorogenic acid standard.

### 2.3. Flavonoid Content of the AE

The quantification of total flavonoid content was undertaken as previously described [25]. So, 0.5 mL of extract was added to 1 mL of the aluminum chloride reagent, and then the color was allowed to develop for 30 min. Afterward, the absorbance was recorded at 430 nm, and the concentrations were determined using a calibration curve of rutin.

### 2.4. HPLC–DAD Analysis of the AE

The HPLC–DAD profiling was undertaken as previously reported [26]. Briefly, 10 µL of the AE were filtered through a 0.45 µm filter and injected into a C18 column (250 × 4.6 mm, 5 µm). The compounds were separated using a mobile phase composed of acidified ultrapure water (A) and methanol (B) as follows: 20 min: 20% B; 15 min: 100% B; 5 min: 20% B. The flow rate of the mobile phase was 1 mL/min, at temperature of 20 °C, and peaks were recorded at 340 nm. The compound identification was based on UV–visible spectra and retention times according to a database of standard compounds. The quantitative analysis was carried out using external calibration.

### 2.5. Animals and Treatments

#### 2.5.1. Elaboration of the High-Fat High-Sucrose Diet (HFSD)

The HFSD was formulated in the laboratory according to our previous method slightly modified [26] Thus, a standard mice diet (society “Provimac-Morocco”) was mixed with lard (16%), cholesterol (1.5%), egg yolk (10%), and sucrose (10%).

#### 2.5.2. Experimental Schedule

Adult male albino mice weighing 24–26 g bred in the animal house of the Faculty of Sciences, (University Mohamed I, Oujda, Morocco) were provided free access to diet and water ad libitum. The temperature in the animal house was maintained at 22 °C with a 12 h light–dark cycle. The animal experiments adhered to the Care and Use of Laboratory Animals Guidelines set forth by the US National Institutes of Health (NIH Publication No. 85-23, revised 1996) and received approval from the local animal use committee (approval number: CS.952023) issued on 2 January 2023.

The mice were assigned randomly into 5 groups, with each group consisting of eight animals. The normal group (NG) was kept on a standard diet and gavaged with distilled water. The hyperlipidemic group (HG) was kept on HFSD and gavaged with distilled water. The aqueous artichoke bract extract (AE) treated group (AETG) was kept on HFSD and gavaged with AE at 100 and 200 mg/kg BW/day. The fenofibrate group (FG) was kept on HFSD and treated with fenofibrate at 4 mg/kg.

Food intake was measured daily, and body weight weekly during the period of treatment (8 weeks). The blood was taken from mice retro-orbital sinus under sodium citrate and immediately centrifuged at 2500 rpm/10 min. The plasma was used for biochemical analysis. At the end of the experiment, the liver and abdominal fat were excised and immediately washed in cold aqueous NaCl solution (0.9%). Fecal samples were also collected after 4 and 8 weeks of the experiment.

#### 2.5.3. Biochemical Analyses of Plasma

Triglycerides, total cholesterol, LDL–cholesterol, HDL–cholesterol, glucose, alanine aminotransferase (ALT), aspartate aminotransferase (AST), and alkaline phosphatase (ALP) were analyzed using specific biomedical kits.

### 2.6. Liver, Fecal, and Biliary Lipid Analysis

After extraction of lipids as previously outlined [27], total cholesterol and triglycerides in liver and feces were determined using enzymatic methods. Biliary cholesterol was measured by specific biomedical kits after recuperation of bile.

### 2.7. Measurement of Hepatic Oxidative Stress: MDA, Glutathione, SOD, and Catalase Enzymes

MDA was determined spectrophotometrically after reaction with thiobarbituric acid according to the method of Mokhtari et al. [28]. Glutathione (GSH) amount was measured using the method of Ellman based on the reaction with 5, 5-dithiobis-(2-nitrobenzoic acid) [29]. The activities of liver enzymes, SOD, and CAT, were assessed using our previously published method [30].

### 2.8. Histology of Liver

Small pieces of fresh liver from all treated mice were fixed in buffered formalin (10%) and embedded in paraffin before being cut and stained with hematoxylin and eosin. The sections were observed using an optical microscope [31].

### 2.9. Acute Toxicity of the AE

The acute toxicity evaluation of the AE was carried out as described in OECD Guideline 425 [32]. Adult *Albino* mice of two sexes weighing 27–30 g were fasted overnight, divided into groups of 4 animals each (2 males and 2 females) in individual cages, and gavaged with the AE at increasing doses (500, 1000, 2000, and 5000 mg/kg). The animals were observed for mortality and other morphologic symptoms of toxicity over 2 weeks.

### 2.10. Molecular Docking Analysis

The in silico investigation was carried out with the SwissDock version: 2023 program (https://www.swissdock.ch/ (accessed on 20 September 2023)). Protein structures were collected from the UniProt Data Bank (https://www.uniprot.org (accessed on 20 September 2023)), whereas chemical structures of polyphenols were retrieved from the PubChem database (https://pubchem.ncbi.nlm.nih.gov (accessed on 20 September 2023)). The binding interactions were studied for the following targets: PPARα (Peroxisome Proliferator-Activated Receptor Alpha); LXRα (Liver X Receptor Alpha); SREBP-2 (Sterol Regulatory Element-Binding Protein 2); PCSK-9 (Proprotein Convertase Subtilisin/Kexin Type 9); NF-κB (Nuclear Factor kappa-B). The binding affinities were measured in kcal/mol, with values below −7 kcal/mol indicating probable interactions.

### 2.11. HepG2 Cells Culture and Western Blot Analysis

The culture of human HepG2 cells and protein extraction were conducted following the protocol outlined by Liu et al. [33]. In summary, HepG2 cells were maintained in Dulbecco’s Modified Eagle Medium (DMEM) (Sigma Aldrich, St. Louis, MO, USA) enriched with 10% fetal bovine serum and 1% penicillin–streptomycin, and incubated at 37 °C in a 5% CO_2_ atmosphere. When the cultures reached approximately 80% confluency, they were treated with trypsin and replenished. The cells were then seeded into culture plates at a density of 106 cells per well and allowed to adhere for 24 h. The experimental treatment involved the following groups: cells treated with PBS (control), cells treated with 1,5-di-*O*-caffeoylquinic acid at concentrations of 0.15, 0.30, and 0.45 µg/mL; cells treated with chlorogenic acid at 0.15, 0.30, and 0.45 µg/mL; cells treated with 1,3-di-*O*-caffeoylquinic acid at 0.15, 0.30, and 0.45 µg/mL; cells treated with luteolin at 0.15, 0.30, and 0.45 µg/mL; and cells treated with the AE at 0.45 µg/mL. After 24 h, proteins were extracted from the cells, separated by SDS-PAGE, and subsequently transferred onto a polyvinylidene difluoride membrane. The membranes were then blocked using bovine serum and incubated overnight with primary antibodies against PCSK-9, Apo A-1, SREBP-2, CYP2E1, HMG-CoA reductase, and p-AMPK (all from Abcam, Cambridge, MA, USA). The blots were washed and incubated with secondary antibodies for 2 h.

### 2.12. Cells Viability Assay

HepG2 cells were cultured and exposed to different concentrations of 1,5-di-*O*-caffeoylquinic acid, chlorogenic acid, 1,3-di-*O*-caffeoylquinic acid, luteolin, and the AE (0.25−2.5 µg/mL) for 72 h. Cell viability was assessed using the MTT reduction assay, where the absorbance of formazan, a product of the reduction process, was measured at 570 nm.

### 2.13. Statistical Analysis

Data were analyzed using one-way analysis of variance (ANOVA). Differences with *p*-values below 0.05 were deemed statistically significant. Results are presented as mean ± SEM.

## 3. Results

### 3.1. Phenolic Composition of the AE

Analysis of the major phenolic classes in the aqueous artichoke bract extract (AE) reveals that it contains a high quantity of total phenols (353.57 ± 3.09 mg/g). Flavonoids accounted for 190.42 ± 1.16 mg/g, with the remainder presumably consisting of phenolic acids and other components. HPLC analysis showed that the AE does indeed contain a mixture of phenolic acids and flavonoids, including 1,5-di-*O*-caffeoylquinic acid (105.93 mg/g), chlorogenic acid (70.62 mg/g), 1,3-di-*O*-caffeoylquinic acid (35.31 mg/g), and luteolin (17.65 mg/g) (Table 1).

### 3.2. Effect of AE on Body Weight and Food Intake

**Figure 1:**
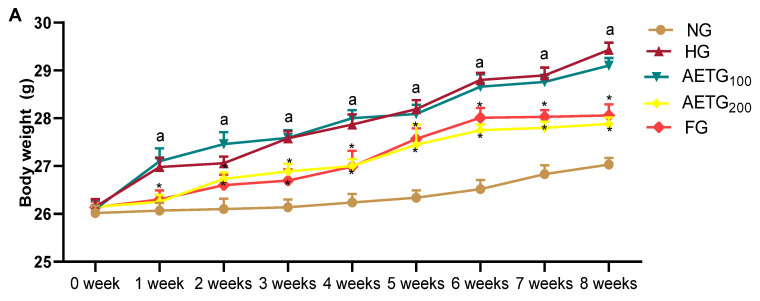

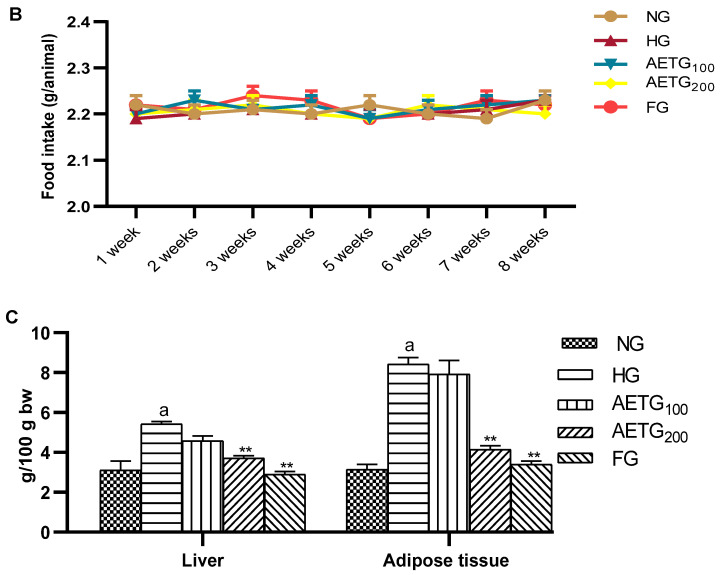
Effect of AE on body weight (**A**), food intake (**B**), and organ relative weight in mice (**C**). AE: aqueous artichoke bract extract; NG: normal group; HG: hyperlipidemic group; AETG: AE-treated groups; FG: fenofibrate group. ^a^ *p* < 0.001 vs. NG. * *p* < 0.01 and ** *p* < 0.001 vs. HG.

The body weight of animals in all groups increased significantly from the beginning to the end of the experiment. In fact, the body weight gain in NG was 3% (*p* < 0.01), in HG of 12% (*p* < 0.01), in AETG 100 of 10% (*p* < 0.01), in AETG200 of 6% (*p* < 0.01), and in FG of 5.8% (*p* < 0.01). Furthermore, at the end of the experiment, we observed that the HFSD resulted in a significant increase in mice body weight in HG compared to NG (*p* < 0.01). However, the AE at 200 mg/kg and fenofibrate exerted significant body weight loss of 7% and 6% (*p* < 0.01), respectively. We also note that the AE at the lower dose (100 mg/kg) did not induce any significant changes in mice body weight (Figure 1A).

Additionally, no significant differences were observed in food intake between all treated groups, suggesting that AE, fenofibrate, and HFSD did not affect the appetite of mice (Figure 1B). However, as shown in Figure 1C, the liver and adipose tissue weights of the HFSD-fed mice were significantly higher than those of the control, with increases of 70% (*p* < 0.001) and 20% (*p* < 0.001), respectively. Treatment with AE at 100 mg/kg did not produce any significant changes in these parameters. However, the higher dose of AE led to a 43% (*p* < 0.001) reduction in liver weight and a 63% (*p* < 0.001) reduction in adipose tissue weight. Fenofibrate also had a similar impact, decreasing liver weight by 46% (*p* < 0.001) and adipose tissue weight by 59% (*p* < 0.001).

### 3.3. Effect of AE on Plasma Lipid Levels

**Figure 2:**
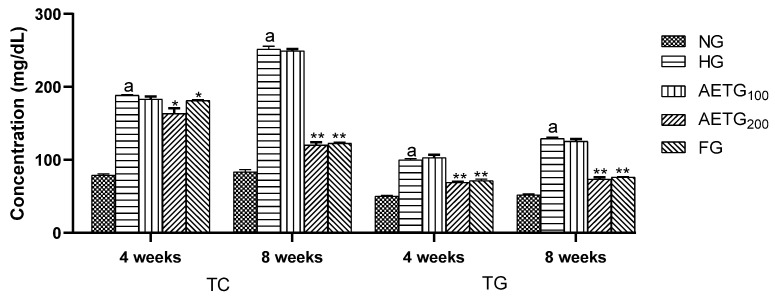
Effect of AE on plasma total cholesterol and triglyceride levels in hyperlipidemic mice. AE: aqueous artichoke bract extract; TC: total cholesterol; TG: triglycerides; NG: normal group; HG: hyperlipidemic group; AETG: AE-treated groups; FG: fenofibrate group. ^a^ *p* < 0.001 vs. NG. * *p* < 0.01 and ** *p* < 0.001 vs. HG.

**Figure 3:**
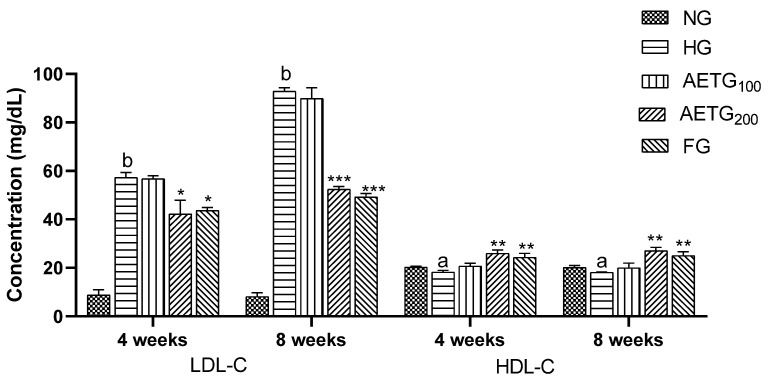
Effect of AE on plasma LDL–C and HDL–C levels in mice. AE: aqueous artichoke bract extract; HDL–C: high-density lipoprotein-cholesterol; LDL–C: low-density lipoprotein-cholesterol; NG: normal group; HG: hyperlipidemic group; AETG: AE-treated groups; FG: fenofibrate group. ^a^ *p* < 0.05 and ^b^ *p* < 0.001 vs. NG. * *p* < 0.05, ** *p* < 0.01, and *** *p* < 0.001 vs. HG.

The effects of AE on plasma lipids are summarized in Figure 2 and Figure 3. After 4 weeks, in the hyperlipidemic control group, significant increases were observed in TC (66.6%, *p* < 0.001), TG (36.9%, *p* < 0.001), and LDL–C (74.8%, *p* < 0.001). After 8 weeks, these levels continued to increase, with a 96.8% (*p* < 0.001) rise in TC, 56.8% (*p* < 0.001) in TG, and 87.1% (*p* < 0.001) in LDL–C. Moreover, the HDL–C decreased by 6.3% (*p* < 0.05) after 4 weeks and 8.5% (*p* < 0.05) after 8 weeks.

However, AE at higher doses had a pronounced impact on plasma lipid profile. Thus, after 4 weeks, TC decreased by 13.3% (*p* < 0.01), TG by 30.8% (*p* < 0.001), and LDL–C by 15% (*p* < 0.05) compared to the HG group. While, after 8 weeks, the reductions were more substantial, so the extract decreased total cholesterol, TG, and LDL–C by 52.6%, 43.6%, and 45.3%, respectively (*p* < 0.001). In addition, the AE increased HDL–C by 20% (*p* < 0.01) after 4 weeks and by 23.5% (*p* < 0.001) after 8 weeks.

The fenofibrate resulted in significant reductions of TC (34.4%, *p* < 0.01), TG (28.3%, *p* < 0.001), and LDL–C by 16%, (*p* < 0.05) after 4 weeks. These changes continued to rise after 8 weeks so that TC reduced by 51.4% (*p* < 0.001), TG by 43.6% (*p* < 0.001), and LDL–C by 45.3% (*p* < 0.01), while HDL–C increased by 25.3% (*p* < 0.01).

It is worth noting also that the AE at a lower dose (100 mg/kg) did not exert any significant effect on the plasma lipid profile.

### 3.4. Effect of AE on Liver and Adipose Tissue Lipid Levels

**Figure 4:**
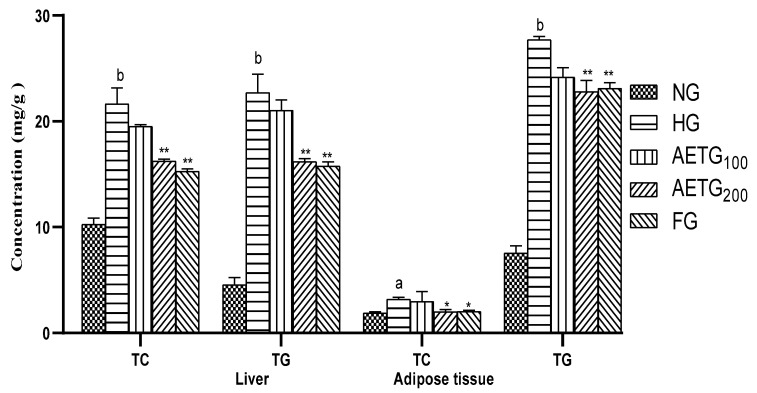
Effect of AE on lipid levels in the liver and adipose tissue of mice. AE: aqueous artichoke bract extract; TC: total cholesterol; TG: triglycerides; NG: normal group; HG: hyperlipidemic group; AETG: AE-treated groups; FG: fenofibrate group. ^a^ *p* < 0.01 and ^b^ *p* < 0.001 vs. NG. * *p* < 0.05 and ** *p* < 0.001 vs. HG.

The data on the effect of HFSD and AE on liver and adipose tissue lipids were summarized in Figure 4. As can be seen, in the liver, the HFSD significantly elevated TC levels by 52% (*p* < 0.001) and TG by 86.59% (*p* < 0.001), while AE at 200 mg/kg prevents this increase by 24.98% (*p* < 0.001) for TC and 28.65% (*p* < 0.001) for TG. The fenofibrate decreased these parameters by 29.43% (*p* < 0.001) and 30.62% (*p* < 0.001), respectively.

In the adipose tissue, the same evolution was observed so that the HFSD increased TC by 49% (*p* < 0.01) and TG by 198% (*p* < 0.001). The AE at 200 mg/kg significantly corrected these disorders, reducing the TC by 36.68% (*p* < 0.05) and TG by 17.72% (*p* < 0.001). These decreases were comparable to those observed in the fenofibrate group, which showed a 37.74% (*p* < 0.05) reduction in TC and 16.54% (*p* < 0.001) in TG. Finally, we note that the effect of AE at 100 mg/kg remains not significant.

### 3.5. Effect of AE on Biliary Cholesterol

**Figure 5:**
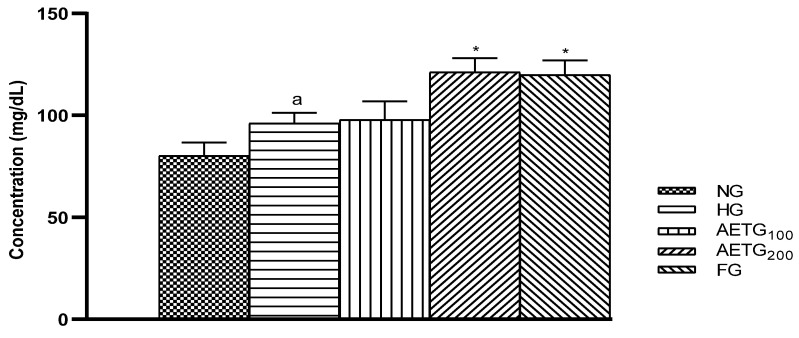
Effect of AE on biliary cholesterol. AE: aqueous artichoke bract extract; NG: normal group; HG: hyperlipidemic group; AETG: AE-treated groups; FG: fenofibrate group. ^a^ *p* < 0.05 vs. NG. * *p* < 0.01 vs. HG.

Figure 5 depicts the changes in biliary cholesterol levels across treated groups. Mice on HFSD alone for 8 weeks exerted a moderate increase in biliary cholesterol excretion, rising by 32% (*p* < 0.05) compared to the normal control group, whereas the AE at 200 mg/kg increased this excretion by 85% (*p* < 0.01) compared to the hyperlipidemic group. As for the fenofibrate drug, it raised biliary cholesterol excretion by 84% (*p* < 0.01). The AE at 100 mg/kg produced no significant effect on this parameter.

### 3.6. Effect of AE on Fecal Lipid Excretion

**Figure 6:**
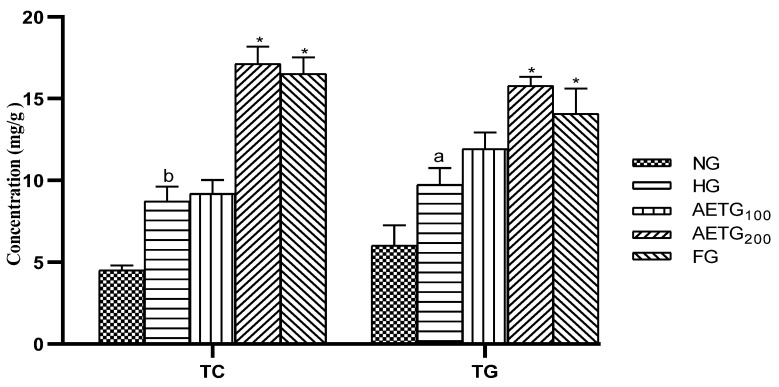
Effect of AE on fecal lipid excretion in mice. AE: aqueous artichoke bract extract; TC: total cholesterol; TG: triglycerides; NG: normal group; HG: hyperlipidemic group; AETG: AE-treated groups; FG: fenofibrate group. ^a^ *p* < 0.01 and ^b^ *p* < 0.001 vs. NG. * *p* < 0.001 vs. HG.

Mice on HFSD exhibited a significant increase in TC (33%, *p* < 0.001) and TG (22%, *p* < 0.01) in their feces compared to control. Administering AE at a higher dose provoked a significant rise in these parameters after 8 weeks. In fact, TC and TG increased by 47% and 48%, respectively (*p* < 0.001). Conversely, AE at 100 mg/kg does not hinder TC and TG excretion. Fenofibrate treatment also enhanced fecal TC elimination by 41% (*p* < 0.001) and TG by 39% (*p* < 0.001) (Figure 6).

### 3.7. Effect of AE on Plasma Glucose Levels

**Figure 7:**
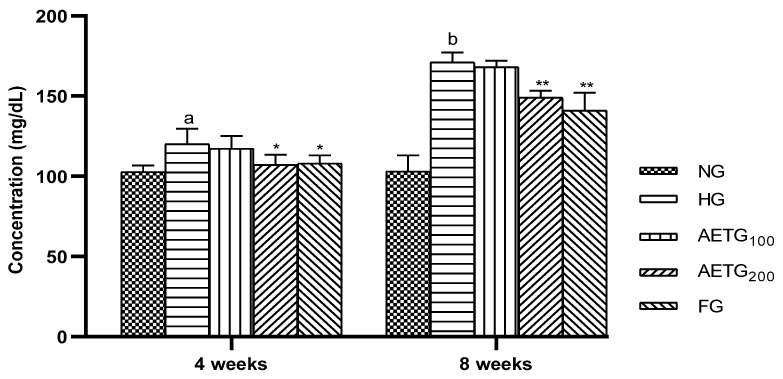
Effect of AE on glucose levels in mice. AE: aqueous artichoke bract extract; NG: normal group; HG: hyperlipidemic group; AETG: AE-treated groups. FG: fenofibrate group. ^a^ *p* < 0.01 and ^b^ *p* < 0.001 vs. NG. * *p* < 0.01 and ** *p* < 0.001 vs. HG.

As we can see, plasma glucose in the HFSD-fed mice rose by 31% (*p* < 0.01), but AE at a higher dose significantly suppressed this rise after 4 weeks (12%, *p* < 0.01) and 8 weeks (17%, *p* < 0.001), respectively. However, at lower doses, the AE exerted no significant effect. As for fenofibrate, it decreased plasma glucose at 4 weeks and 8 weeks by 14% (*p* < 0.01) and 21% (*p* < 0.001), respectively (Figure 7).

### 3.8. Effect of AE on Liver Histology

**Figure 8:**
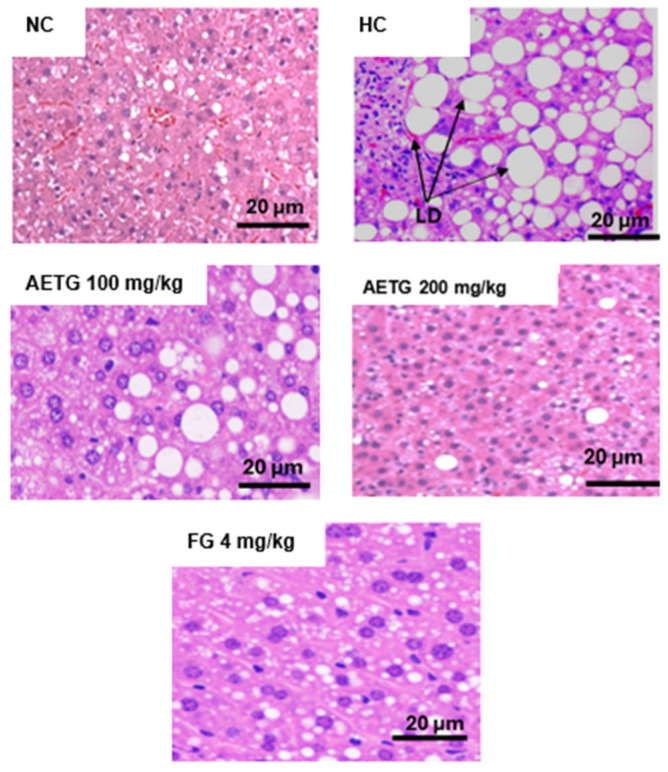
Effects of AE on mice liver histology. AE: aqueous artichoke bract extract; LD: lipid droplets; NG: normal group; HG: hyperlipidemic group; AETG: AE-treated groups; FG: fenofibrate group.

Figure 8 illustrates the effect of AE on liver histology. It is evident that normolipidemic mice exhibited a generally normal liver appearance. In contrast, the liver tissue from the hyperlipidemic group showed important necrosis and increased cytoplasmic lipid deposition. These adverse effects were notably reduced in the groups treated with 200 mg/kg of AE and fenofibrate.

### 3.9. Effect of AE on Transaminase and Phosphatase Alkaline

**Figure 9:**
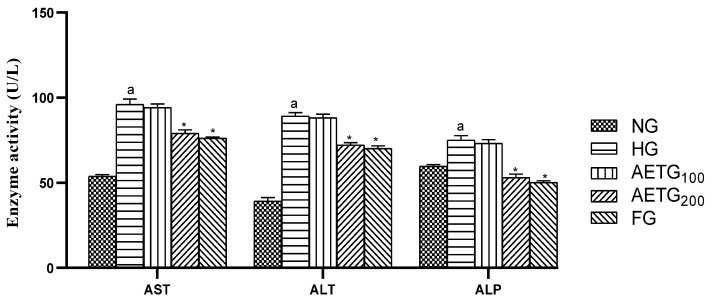
Effect of AE on enzymatic biomarkers of hepatic injury in mice. AE: aqueous artichoke bract extract; AST: aspartate transaminase; ALT: alanine transaminase; ALP: alkaline phosphatase; NG: normal group; HG: hyperlipidemic group; AETG: AE-treated groups; FG: fenofibrate group. ^a^ *p* < 0.001 vs. NG. * *p* < 0.001 vs. HG.

The hyperlipidemic group exhibited significant increases in hepatic injury biomarkers compared to the normal control group. In fact, it shows a rise of 44.00% in AST (*p* < 0.001), 56.08% in ALT (*p* < 0.001), and 20.56% in ALP activity (*p* < 0.001). Administering AE at lower doses resulted in only minor, non-significant changes in these biomarkers. However, the higher dose of 200 mg/kg significantly mitigated these elevations, with reductions of 45.63% for AST (*p* < 0.001), 57.08% for ALT (*p* < 0.001), and 22.66% for ALP (*p* < 0.001). The fenofibrate-treated group also showed notable decreases, with reductions of 20.72% for AST (*p* < 0.001), 21.30% for ALT (*p* < 0.001), and 33.23% for ALP (*p* < 0.001) (Figure 9).

### 3.10. Effect of AE on Liver MDA in Hyperlipidemic Mice

**Figure 10:**
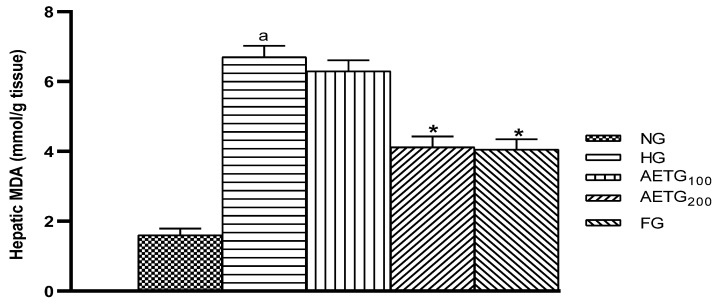
Effect of AE on liver lipid oxidation in mice. AE: aqueous artichoke bract extract; NG: normal group; HG: hyperlipidemic group; AETG: AE-treated groups; FG: fenofibrate group. ^a^ *p* < 0.001 vs. NG. * *p* < 0.001 vs. HG.

The HFSD induced a significant increase in hepatic MDA levels (76%, *p* < 0.001). This increase was corrected by the concomitant consumption of AE at a dose of 200 mg/kg, resulting in a 38% (*p* < 0.001) reduction, while the 100 mg/kg dose was found to be non-significant. Fenofibrate decreased this parameter by 39.10% (*p* < 0.001) (Figure 10).

### 3.11. Effect of AE on Glutathione, SOD, and Catalase

**Figure 11:**
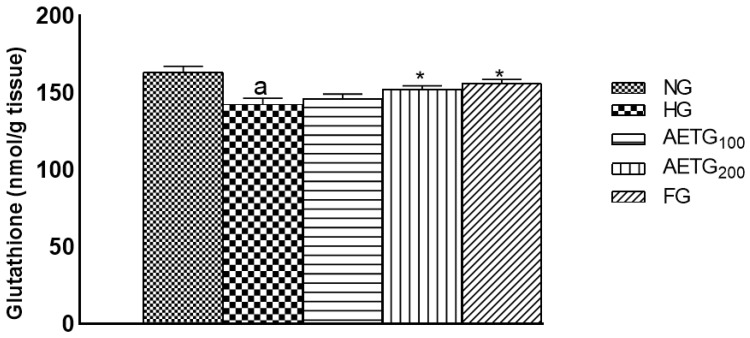
Effect of AE on glutathione in hyperlipidemic mice. AE: aqueous artichoke bract extract; NG: normal control group; HG: hyperlipidemic group; AETG: AE-treated groups; FG: fenofibrate group. ^a^ *p* < 0.01 vs. NG. * *p* < 0.01 and vs. HG.

**Figure 12:**
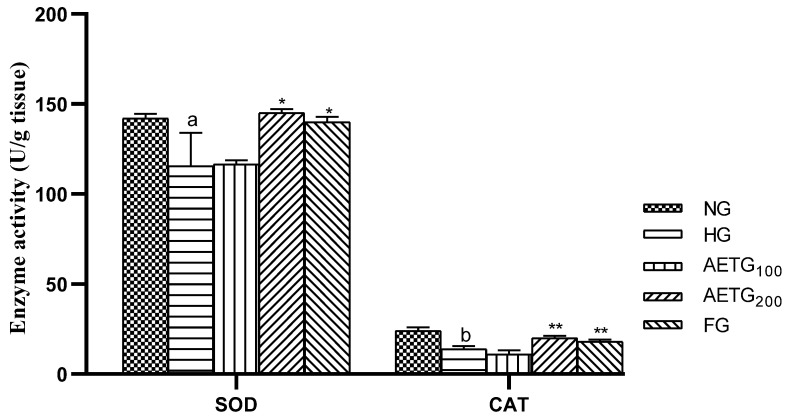
Effect of AE on superoxide dismutase and catalase activities in hyperlipidemic mice. AE: aqueous artichoke bract extract; NG: normal group; HG: hyperlipidemic group; AETG: AE-treated groups; FG: fenofibrate-treated group. ^a^ *p* < 0.01 and ^b^ *p* < 0.001 vs. NG. * *p* < 0.01 and ** *p* < 0.001 vs. HG.

The obtained results show that the HFSD exerted a significant decrease in the glutathione level (10%, *p* < 0.01) and activity rate of the enzymes SOD (22.94%, *p* < 0.01) and catalase (76%, *p* < 0.001), thus indicating a microenvironment of oxidative stress. In contrast, AE at 200 mg/kg increased glutathione by 32% and enhanced SOD activity by 25% (*p* < 0.01) and catalase activity by 44% (*p* < 0.001) (Figure 11 and Figure 12). We note also that the 100 mg/kg dose of AE did not significantly affect glutathione content and enzyme activities.

### 3.12. In Silico Interaction Between AE Phenolic Compounds and Principal Protein Implicated in Lipid Metabolism and Oxidative Stress

The binding affinities, reported in kcal/mol, between the main polyphenols found in artichoke extract and proteins implicated in oxidative stress and lipid metabolism are summarized in Table 2. The results show that 1,5-di-*O*-caffeoylquinic acid has a strong interaction with SREBP-2 and PCSK-9. Furthermore, chlorogenic acid strongly binds to PPARα and LXRα. Luteolin appears to interact with NF-κB, a protein linked to oxidative stress and inflammatory processes. This suggested that these compounds could be a candidate for regulating lipid disorders and decreasing hepatic steatosis and oxidative stress.

### 3.13. Effects of AE and Its Major Identified Polyphenols on SREBP-2, HMG-CoA-R, PCSK-9, Apo A-1, p-AMPK, and CYP2E1 Expression in HepG2 Cells

**Figure 13:**
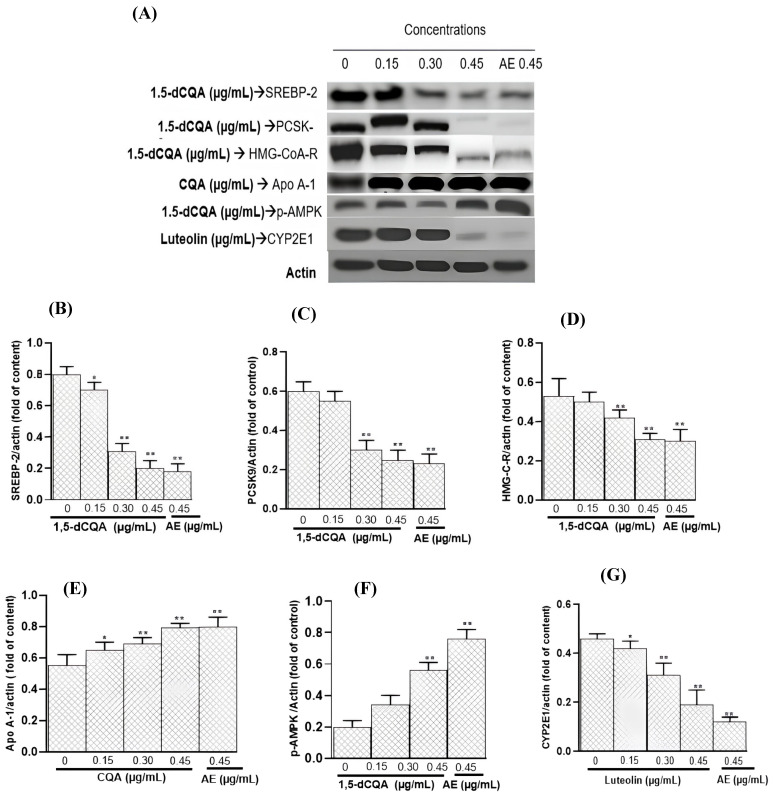
Effects of AE and its major identified polyphenols on protein expression in HepG2 cells. 1,5-dCQA: 1,5-di-*O*-caffeoylquinic; CQA: chlorogenic acid; AE: aqueous artichoke bract extract; PCSK-9: proprotein convertase subtilisin/kexin type 9; Apo A-1: apolipoprotein A-1; SREBP-2: sterol regulatory element-binding protein 2; CYP2E1: cytochrome P450 2E1; HMG-C-R: 3-hydroxy-3-methyl-glutaryl-CoA reductase; p-AMPK: phosphorylated AMP-activated protein kinase. * *p* < 0.01, ** *p* < 0.001 vs. control (0 µg/mL).

The Western blot analysis revealed significant variations in the expression levels of SREBP-2, HMG-CoA-R, PCSK-9, Apo A-1, p-AMPK, and CYP2E1 proteins in HepG2 cells treated with AE and different standard polyphenols identified within it, including 1,5-di-*O*-caffeoylquinic, chlorogenic acid, and luteolin at concentrations of 0.15, 0.30, and 0.45 µg/mL (Figure 13A). It is worth noting that AE treatment resulted in a reduction in SREBP-2, PCSK-9, HMG-CoA-R, and CYP2E1 expressions, as well as an increase in p-AMPK, which could indicate a potential influence on lipid metabolism and oxidative stress regulation pathways. Specifically, the polyphenols’ effects both confirmed and expanded upon the results observed with AE. The expression of SREBP-2, PCSK-9, and HMG-CoA-R decreased progressively with increasing concentrations of 1,5-di-*O*-caffeoylquinic compared to the control (PBS) (Figure 13B–D). Regarding Apo A-1, the results showed a notable increase in its expression in cells treated with chlorogenic acid, particularly at the highest concentration of 0.45 µg/mL, suggesting a potential induction of this protein involved in the reverse cholesterol transport pathway (Figure 13E). The expression of p-AMPK, a key regulator of energy metabolism, was significantly upregulated in cells treated with 1,5-di-*O*-caffeoylquinic at 0.15, 0.30, and 0.45 µg/mL, indicating a stronger activation of p-AMPK-associated metabolic pathways (Figure 13F). The CYP2E1, implicated in liver management of oxidative stress and steatosis, was significantly downregulated by luteolin, indicating that this flavonoid could be behind the antioxidant and antisteatotic effects of the AE (Figure 13G). The expression of the loading control protein, actin, remained consistent across all samples, confirming equal protein loading and the reliability of the results.

### 3.14. Acute Toxicity and HepG2 Cells Viability

**Figure 14:**
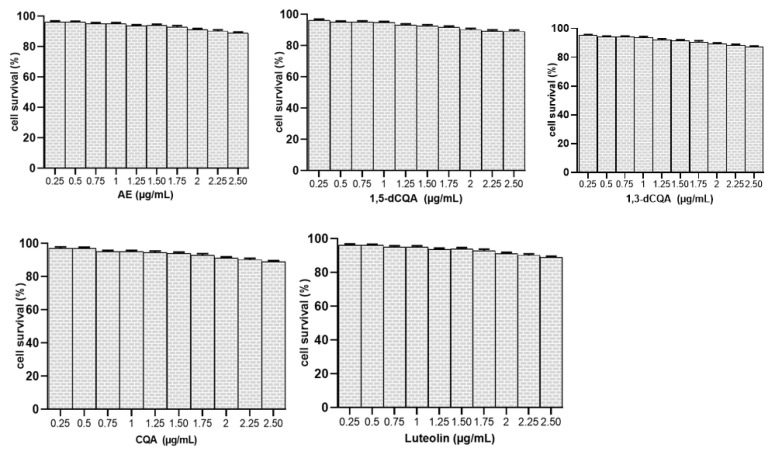
Effects of AE and its major identified polyphenols on HepG2 cell viability. 1,5-dCQA: 1,5-di-*O*-caffeoylquinic; CQA: chlorogenic acid; AE: aqueous artichoke bract extract.

The acute toxicity study showed that during the 14-day observation period, no signs of toxicity appeared in animals treated with doses ranging from 500 up to 5000 mg/kg BW. This suggests that the AE could be classified as non-toxic with an LD50 value greater than 5000 mg/kg. Additionally, the viability of HepG2 cells was not substantially impacted by the AE or any of its phenolic components (Figure 14).

## 4. Discussion

It is well-recognized that the majority of metabolic and cardiovascular disorders are associated with the frequent consumption of diets rich in fat and sugar [34]. Worldwide, a growing number of people are using herbal remedies to treat hyperlipidemia and related complications [35]. In Morocco, artichoke (*Cynara scolymus* L.) is a medicinal plant commonly used as a treatment for hyperlipidemia [18]. Nevertheless, there is currently little experimental research available on this topic. In this study, we demonstrated the effects of aqueous artichoke bract extract (AE) on lipid metabolism disturbances and related hepatic complications while highlighting the implication of major identified phenolic compounds in underlying mechanisms.

The primary outcome of this investigation is that the high-fat, high-sucrose diet impairs multiple mice metabolic parameters in plasma and liver, leading to obesity, hyperlipidemia, hyperglycemia, hepatic steatosis, and oxidative stress. However, the treatment with the AE significantly corrected these imbalances. So in what follows, we will discuss all the significant metabolic effects through the possible involvement of identified polyphenols in the regulation of hindered metabolic pathways. This will be conducted by linking the results of the in vivo study to those of the protein–ligand interaction in silico and the mechanisms highlighted in HepG2 cells in vitro.

As can be seen, the AE restored plasma total cholesterol while increasing the anti-atherogenic HDL fraction, ensuring reverse cholesterol transport (RCT) from macrophage and peripheral tissues to the liver for further elimination in bile. This suggests that the phenolic compounds in AE could enhance the cholesterol efflux pathway. This hypothesis was consolidated by the fact that the AE also reduced cholesterol in the liver while concentrating it in the bile for subsequent elimination in the intestine and then in feces. Such a mechanism has also been discussed in previous studies on the hypocholesterolemic effect of artichoke leaves [36,37]. Thus, it is now well known that activation of the RCT pathway is dependent on the expression of apolipoprotein A1 (Apo-A1), which activates LCAT, enabling cholesterol to be purified from macrophages and other peripheral tissues to the liver [38]. So, from the results of molecular docking, only chlorogenic acid, contained in the AE, showed a strong capacity to bind to PPARα and LXRα which are directly implicated in the regulation of Apo A1 gene expression. This suggested that chlorogenic acid could be a candidate in the regulation of the RTC pathway via activating the expression of Apo A1. The hypothesis was validated by the results obtained in HepG2 cells demonstrating that the AE and chlorogenic acid significantly upregulated the expression of Apo A1. This mechanism was proposed by other reports studying the effect of natural compounds, other than artichoke, on cholesterol metabolism [39].

On the other hand, we observed that the modulation of plasma total cholesterol by the AE was accompanied by a significant decrease in the atherogenic LDL fraction, indicating that the phytochemicals contained in this extract could activate the uptake of LDL–cholesterol by peripheral tissues and liver, via its specific receptor (LDL-R) as outlined in other studies [40]. To shed more light on the mechanism that may be involved, we measured the binding force between the major phenolic compounds contained in the extract and the proteins implicated in the regulation of LDL–cholesterol catabolism, mainly SREBP-2, and PCSK-9. So, we realized that only 1,5-di-*O*-caffeoylquinic acid binds firmly to these proteins, suggesting its possible regulatory effect on LDL–cholesterol catabolism. This suggestion was confirmed by measuring the expression of these target proteins in HepG2 cells exposed to AE and its main phenolic compounds. In fact, it was shown that AE and only 1,5-di-*O*-caffeoylquinic acid significantly increased the expression level of PCSK-9 and its activating transcription factor SREBP-2, suggesting its strong involvement in the regulation of LDL–cholesterol catabolism and therefore its possible anti-atherogenic effect. This mechanism was not previously discussed for 1,5-di-*O*-caffeoylquinic acid, while chlorogenic acid from artichoke leaves was shown to activate SREBP-2 [41]. More importantly, the effect of AE on cholesterol homeostasis could also be explained by the possible effect of its phenolic constituents on liver cholesterol endogenous bio-synthesis catalyzed by HMG-CoA reductase as documented in a previous report on artichoke leaves [37]. This hypothesis was built on the basis of the preliminary results of the molecular docking study showing that the 1,5-di-*O*-caffeoylquinic acid exhibited strong binding capacity with SREBP-2, which was known as a transcription factor of the HMG-CoA reductase gene. This observation was confirmed by Western blot results showing that AE and 1,5-di-*O*-caffeoylquinic acid decreased both SERBP-2 and HMG-CoA reductase expression, indicating that this phenolic acid may be the main candidate for modulation of liver endogenous cholesterol biosynthesis by the AE. In the same vein, several reports have highlighted the involvement of chlorogenic acid and luteolin from artichoke leaves in regulating the activity of this enzyme [41].

Additionally, the AE prevented weight gain and especially abdominal obesity in high-fat, high-sucrose-fed mice. This effect is very probably related to lipid-lowering activity since there is a close relationship between hypertriglyceridemia and abdominal obesity [42]. In addition, the extract reduced the deleterious accumulation of triglycerides in the liver, leading to hepatic steatosis and insulin resistance, which could ultimately result in hyperglycemia. This beneficial effect is evidenced by a reduction in plasma glucose and an improvement in the histological appearance of the livers in treated mice. The mechanisms that may be behind these effects are mainly related to liver energy metabolism in the liver and plasma as proposed in other previous reports [43,44]. So, as mentioned above, the 1,5-di-*O*-caffeoylquinic acid interacted with SREBP-2, in silico, and upregulated the expression of p-AMPK in HepG2 cells, a mechanism by which lipogenesis could be inhibited. This result corroborates with previous studies reporting the effect of artichoke leaf phenolics on AMPK expression [41].

Moreover, hyperlipidemia and fatty liver are generally accompanied by oxidative stress, leading to lipid oxidation and the production of toxic free radicals that cause inflammation and cell damage [45]. In this study, treatment with AE reinforced liver antioxidant defense by increasing glutathione levels and the activities of SOD and CAT, which resulted in a significant decrease in MDA. The extract also decreased the activities of alanine transaminase, aspartate transaminase, and alkaline phosphatase, indicating a significant hepatoprotective effect of the extract against steatosis. This effect could be most likely related to the anti-radical and antioxidant activity of polyphenols contained in AE since these compounds are known for their scavenging activity against free radicals as demonstrated using artichoke leaf extracts [21]. So, to explore the mechanism of this effect, we first studied the in silico interaction between the extract’s polyphenols and the nuclear factor NF-κB, which regulates gene expression of several proteins involved in oxidative stress, including SOD and CYP2E1. Secondly, we measured the level of CYP2E1 enzyme expression in HepG2 cells treated or not with the AE and its major phenolic compounds. This enzyme, belonging to the cytochrome P450 family, is strongly implicated in the development of NALFD through the oxidation of free fatty acids and the excessive production of free radicals [46]. Our result demonstrates that among the phenolic compounds, the luteolin flavonoid exhibited the highest binding capacity with NF-κB in silico and the greatest activation of CYP2E1 expression in HepG2 cells. This allows us to confirm the possible implication of luteolin in the antioxidant effect of AE observed in mice liver. In the same vein, several researchers have shown that flavonoids from artichokes could be capable of limiting oxidative stress and its complications in animal models [22]. The analysis of polyphenols in the studied extract shows that the 1,5 di-*O*-caffeoylquinic acid remains the prominent compound. This finding aligns with that obtained by [23,24], studying the phenolic composition of artichoke bracts from Spain and Germany, respectively. However, some studies showed that chlorogenic acid represents the major compound in the artichoke [47]. Furthermore, other researchers reported that 1,3-di-*O*-dicaffeoylquinic acid (cynarin) is the principal compound [48]. However, Schütz et al. [49] consider that this is an artifact formed by transesterification of 1,5-di-*O*-caffeoylquinic acid through aqueous extraction.

Regarding their beneficial effect on cardiometabolic health, 1,5-di-*O*-caffeoylquinic acid has not yet been studied previously. As for chlorogenic acid, its general health advantages are related to its antioxidant qualities, which are the reason for its broad recognition, whereas underlying mechanisms are little discussed [47]. Concerning flavonoids, derivatives of luteolin and apigenin are the main artichoke compounds reported in the literature [50]. In this study, only luteolin was identified in the aqueous artichoke extract. This compound is mainly discussed for its antioxidant effects and health benefits [50,51].

Finally, we note that all metabolic effects exerted by AE in HFSD-fed mice are very close and comparable to that of fenofibrate, a standard lipid-lowering drug known for its lowering activity on plasma LDL–cholesterol, triglycerides, and glucose according to several mechanisms, most of which have been discussed above [52].

## 5. Conclusions

Based on the findings of this study, we conclude that the aqueous artichoke bract extract contains bioactive polyphenols capable of preventing lipid metabolism disorders, oxidative stress, and fatty liver disease by regulating the expression of key proteins, including HMG-CoA reductase, ApoA-1, SREBP-2, PCSK-9, p-AMPK, and CYP2E1. Notably, this study is the first to establish the potential mechanisms of artichoke bract extract in modulating these specific molecular targets in a hyperlipidemic model, providing a scientific basis for its traditional use in Morocco to treat hyperlipidemia. While our findings partially validate the folk medicinal use of artichoke, clinical studies are needed to further translate these experimental results into a medical context. These insights could pave the way for developing novel therapeutic approaches using artichoke-based treatments for lipid disorders and oxidative stress.

## Figures and Tables

**Table 1:** Individual phenolic compounds content in the AE.

Phenolic Compounds	Time Retention (min)	Content (mg/g)
Chlorogenic acid	7.21	70.62
1,5-di-*O*-caffeoylquinic acid	12.43	105.93
1,3-di-*O*-caffeoylquinic acid	17.96	35.31
Luteolin	25.31	17.65

**Table 2:** Binding forces of AE polyphenols with major proteins implicated in the control of lipid homeostasis and liver antioxidative status.

Proteins	1,5-di-*O*-Caffeoylquinic Acid	Chlorogenic Acid	Luteolin	1,3-di-*O*-Caffeoylquinic Acid
PPARα	−6.16	−10.88	−7.50	−6.99
LXRα	−7.02	−10.70	−7.10	−7.05
SREBP−2	−10.19	−7.10	−7.06	−6.89
PCSK-9	−10.62	−7.05	−7.11	−6.26
NF-κB	−6.71	−6.19	−10.17	−7.37

The binding forces were expressed in kcal/mol. PPARα: peroxisome proliferator-activated receptor alpha; LXRα: liver X receptor alpha; SREBP-2: sterol regulatory element-binding protein 2; PCSK-9: proprotein convertase subtilisin/kexin type 9; NF-κB: nuclear factor Kappa-B.

## Data Availability

The data presented in this study are available in article.
